# Supplementary material for: Polyimide Films Based on β-Cyclodextrin Polyrotaxane with Low Dielectric and Excellent Comprehensive Performance
Source: Polymers (Basel). 2024 Mar 25;16(7):901. doi: 10.3390/polym16070901 (PMC11013264; doi:10.3390/polym16070901)
Supplement: Supplementary file 1 [file polymers-16-00901-s001.zip › polymers-2907655-supplementary.pdf]

Supporting Information

Polyimide Films Based on β-Cyclodextrin Polyrotaxane with Low Dielectric and Excellent Comprehensive Performance

Xuexin Zhang [1,†], Yao Dou [1,2,†], Liqun Liu [1], Meixuan Song [1], Zhenhao Xi [1,2,*], Yisheng Xu [1,*], Weihua Shen [1], and Jie Wang [1,*]

[1] State Key Laboratory of Chemical Engineering, East China University of Science and Technology, 200237 Shanghai, China

[2] Shanghai Key Laboratory of Multiphase Materials Chemical Engineering, East China University of Science and Technology, Shanghai 200237, China

[*] Correspondence: zhhxi@ecust.edu.cn (Z.X.); yshxu@ecust.edu.cn (Y.X.); jiewang2010@ecust.edu.cn (J.W.)

[†] These authors contributed equally to this work.

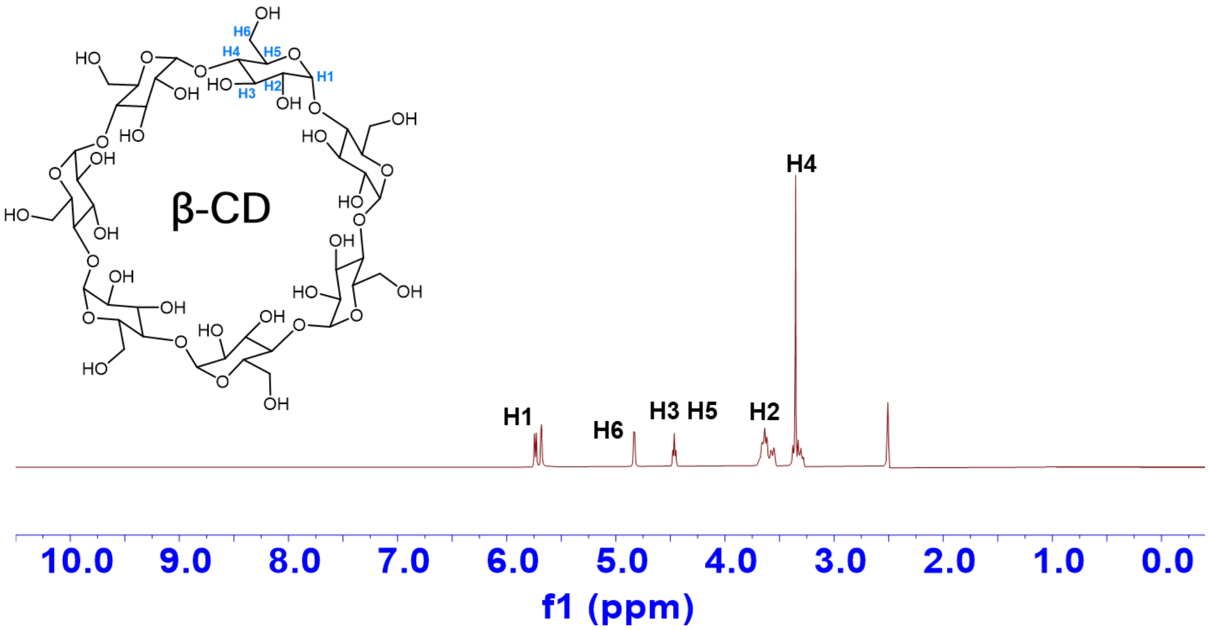

Figure S1: ^{1}H NMR spectra of β-CD (400 MHz, DMSO-d_6) δ 5.74 (d, J = 6.9 Hz, 1H), 5.68 (d, J = 2.5 Hz, 1H), 4.83 (d, J = 3.7 Hz, 1H), 4.46 (t, J = 5.6 Hz, 1H), 3.71 – 3.52 (m, 4H), 3.40 – 3.27 (m, 5H), 2.51 (p, J = 1.8 Hz, 2H).

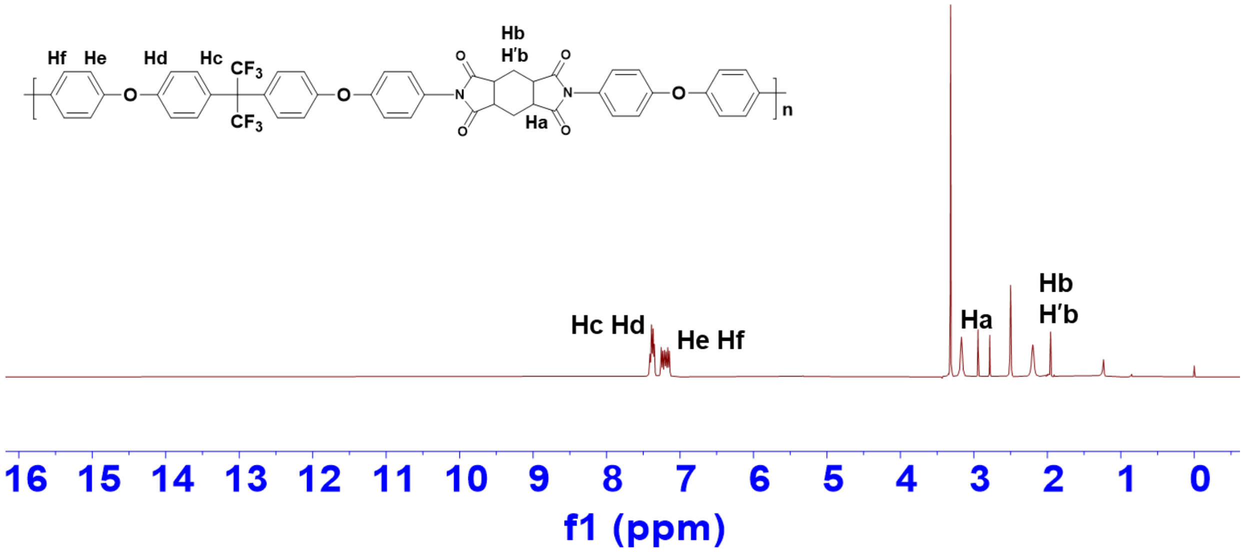

Figure S2: ^{1}H NMR spectra of polyimide (400 MHz, DMSO-d_6) δ 7.43–7.33(m,1H),7.28–7.12(m,1H),3.17(d,J=5.2Hz,1H),2.20(s,1H).

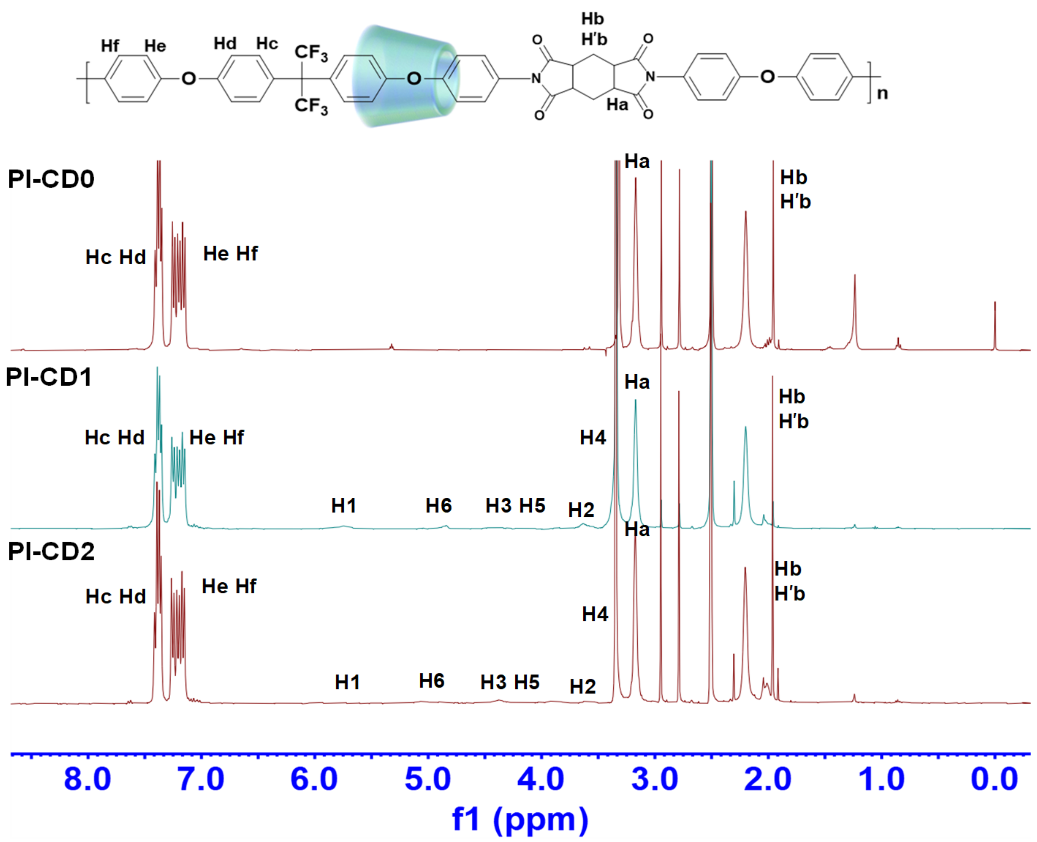

Figure S3: (a): ^{1}H NMR spectra of β-CD/polyimide (600 MHz, DMSO-d_6) δ 7.43–7.34(m, 1H), 7.27–7.05 (m, 1H), 2.94 (s,0H), 2.20 (t, J = 5.2 Hz, 1H). (b): ^{1}H NMR spectra of β-CD/polyimide of the amplified signal.

Table S1: The continuous distance of β-CD/PI calculated by XRD

Sample	2θ	d-space(Å)a
PI-CD0	17.38	5.1013
PI-CD1	16.92	5.2365
PI-CD2	16.82	5.2688
PI-CD3	17.34	5.1114

a: The Bragg equation is used to calculate the continuous spacing

Table S2: GPC results of PI and β-CD/PI composites.

Sample	[η]/（$dL\cdot g^{-1}$）	Mn	Mw	PDI
PI-0	1.84	1.8×10^5	2.6×10^5	1.4487
PI-3	2.07	2.1×10^5	3.0×10^5	1.4384
PI-5	2.76	2.9×10^5	4.0×10^5	1.3877
PI-7	1.79	1.8×10^5	2.6×10^5	1.4681
PI-10	1.52	1.4×10^5	2.2×10^5	1.5409

Table S3: Synthesis of PI and β-CD/PI composites with different β-CD content

Sample	n(β-CD): n（H“PMDA)
PI-CD0	0:1
PI-CD1	1:2
PI-CD2	1:1
PI-CD3	2:1

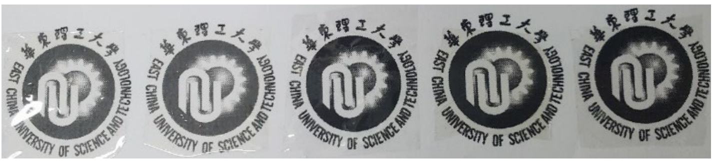

PI-0 PI-3 PI-5 PI-7 PI-10

Figure S4: Photos of PI and β-CD/PI composite films.

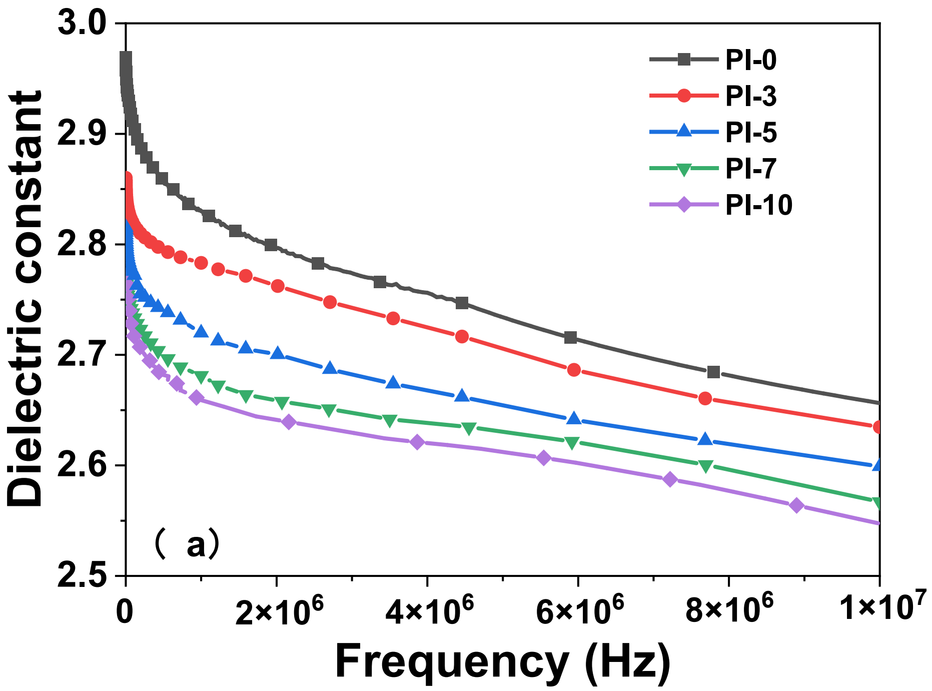

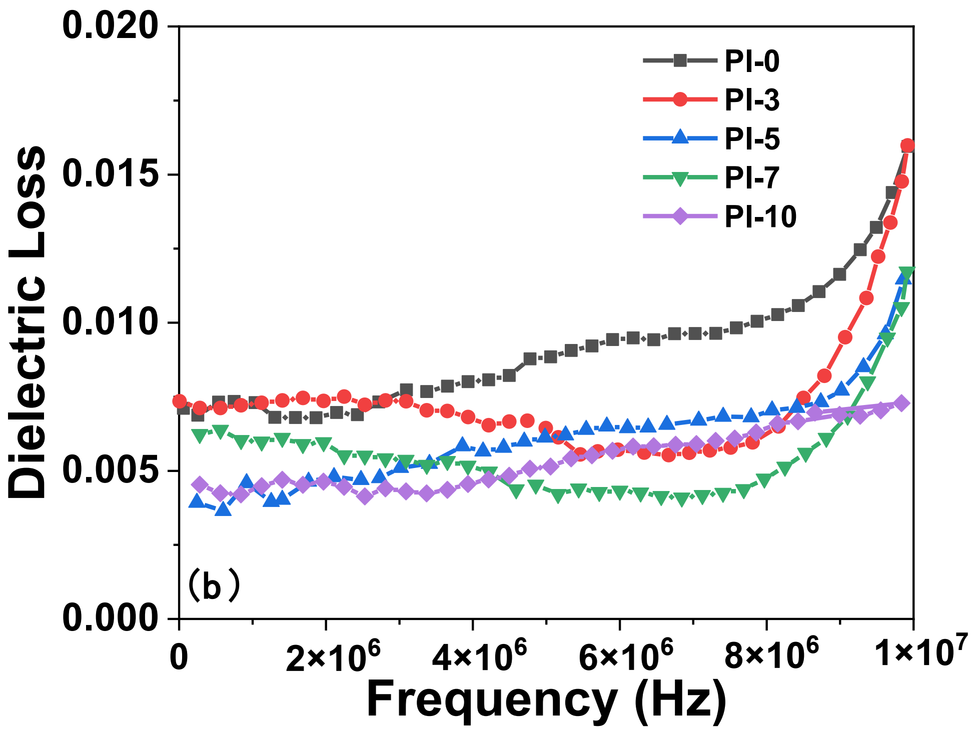

Figure S5: (a): Dielectric constant spectra of PI films without β-CD,
(b): Dielectric loss spectra of PI films without β-CD.

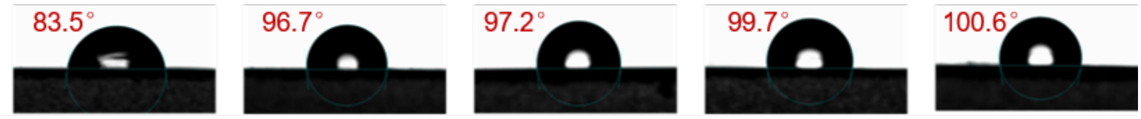

PI-0 PI-3 PI-5 PI-7 PI-10

Figure S6: Water contact angle testing of fluorinated aliphatic copolymer polyimide films.
